# Community Legal Systems: Targeting PrEP and HIV Education to Decrease Risk of HIV Transmission

**DOI:** 10.1007/s10461-021-03219-7

**Published:** 2021-03-19

**Authors:** Leanne Whiteside-Mansell, LaTunja Sockwell, Daniel Knight, Cynthia Crone

**Affiliations:** 1 Department of Family and Community Medicine, College of Medicine, University of Arkansas for Medical Sciences, 521 Jack Stephens Drive #530, Little Rock, AR 72205-7199 USA; 2 College of Public Health, University of Arkansas for Medical Sciences, Little Rock, AR USA

**Keywords:** HIV prevention, PrEP, Drug court, Criminal justice, Stigma

## Abstract

The southern U.S. has both high HIV and incarceration rates in comparison to its population. As in the rest of the country, HIV prevention is based on education, behavior change, and biomedical efforts, such as pre-exposure prophylaxis (PrEP). This study examined the implementation of an educational intervention and supportive services to obtain PrEP in a population of individuals (N = 218) involved in an Adult Drug Court (ADC) or on probation or parole (P-P). Nearly all ADC and P-P participants self-reported risk behaviors linked to HIV acquisition. Results supported the acceptance and usefulness of the intervention as rated by participants. Participants showed increased knowledge of HIV risks and testing post-education. In multivariate analysis, predictors of interest in using PrEP included low stigma beliefs, specifically their level of prejudice views, high depressive symptoms, and white race. The intervention shows promise. Given the high risk documented for ADC and P-P individuals, HIV prevention is a critical component for increased protective behaviors.

## Introduction

While HIV rates have declined over the last 20 years, more than 1.1 million people in the U.S. live with HIV—Human Immunodeficiency Virus [1]. Nationally, the southern U.S. states have a disproportionate burden of transmission, illness, and death related to HIV. This disparity is due to,various reasons, including widespread, chronic poverty, some of the nation's worst health indicators, significant health and social disparities, and an increase in opioid use [2]. Unfortunately, southern states that makeup just 38% of the U.S. population are responsible for 53% of new HIV/AIDS cases in the U.S., 46% of individuals with an HIV diagnosis, and 48% of HIV-related deaths [1, 3, 4]. The high rates are also likely linked to the reaction to perceived stigma (e.g., the highest rates of new cases are among Black men that have sex with men, growing rates among intravenous drug users during the nation’s opioid use epidemic). [5–7].

Beyond the geographic location, HIV risks are high for individuals involved in the legal system, even those in community settings [8–10]. For example, community settings include individuals on probation, parole (P-P), or involved in Adult Drug Courts (ADC). ADCs are specialty courts designed to treat non-violent offenders with substance abuse issues. Individuals on probation or parole from federal, state, or community jails have similar risks as inmates. However, they are at increased risk of exposure to risky sexual behaviors and injection drug use [11]. Less is known about participants of ADCs except that they share many of the demographic factors placing them at risk for HIV [10]. For example, high rates of opioid and other illicit drug use by injection have been reported in ADC populations [12].

Drug courts and criminal justice programs serving individuals in community settings have recently received encouragement for a greater focus on HIV interventions [9, 10, 13]. Further, an array of venues have been proposed as useful in reaching at-risk populations, such as community-based health offices [14–16]; however, embedding HIV education into the required programming may make access most effective. A range of prevention interventions has been promoted to reach adults at risk, including education, HIV testing, and drug treatment [8]. These programs have used educational interventions and HIV testing as the primary methods to reduce risk. Computer-based education programs to reduce HIV risk have resulted in more HIV testing among ADC participants [17]. Access to on-site rapid HIV testing also resulted in more probation and parolees testing [18]. These studies also supported the feasibility and acceptance of the use of HIV-related interventions in these settings [17, 18].

HIV intervention programs, including biomedical prevention options, are increasing; however, research in legal settings is limited [19, 20]. Biomedical efforts such as pre-Exposure Prophylaxis (PrEP) for HIV prevention are one of the cornerstones of the attempt to end the HIV epidemic [21]. Self-administered daily, oral PrEP is an antiretroviral drug prescribed by health care providers to individuals who are HIV negative to protect themselves from contracting HIV when taken consistently. For example, both the iPrEx trial and the Partners PrEP trial showed promise in reducing HIV risk [22, 23].

PrEP for HIV prevention is consistent with the national goals of Healthy People 2020 [24]. The Center for Disease Control (CDC) reports that HIV prevention will require “combinations of scientifically proven, cost-effective, and scalable interventions targeted to the right populations in the right geographic areas” [25]. The CDC lists southern states as a priority for HIV prevention efforts. The high rate of new HIV diagnoses and the low rate of PrEP use in the U.S. South are reasons to be concerned [26, 27]. A targeted focus of the CDC is to increase PrEP use among people without an HIV diagnosis who are at risk for getting the virus from sex or injection drug use. The CDC recommends the use of PrEP in the following individuals who: (1) have a sexual partner with HIV, (2) inconsistently use condoms, (3) recently were diagnosed with a sexually transmitted infection (STI), (4) have a partner that is an injection drug user, or (5) share injection drug use equipment [28].

While the proportion of new HIV diagnoses in the south is larger than all other areas of the country combined, the use of PrEP is low [29, 30]. To put this into perspective, in 2015, a comparison of the rate of HIV diagnoses to prescribed PrEP in the western U.S. was 19% to 27% (HIV/PrEP) and in the southern U.S. is 51% compared to 29.7% [31]. The rate of PrEP use varies nationally by gender and race, with women (4.7% prescribed compared to 95.3% of men) and minorities least likely to have filled a PrEP prescription [32]. Given that the south has the largest proportion of Black Americans as a percentage of its total population, race is likely a factor in the high rate of HIV diagnoses in the south [33]. Nationally, among PrEP users, an estimated 68.7% are white, and only 11.2% are black, and 13.1% are Hispanic. Another group with a high risk of HIV and low use of PrEP are men who have sex with men (MSM), particularly among men from minority racial groups. One barrier seems to have been a lack of awareness given the documented increase in awareness and the concurrent increase in reported use of PrEP from 2014 to 2017 [30, 34]. Still, the combination of minority status and living in the south are linked to low use of PrEP [35].

Little direct research has been conducted to clarify the reasons for the low rates of PrEP use in the south [36]. It is potentially related to the fact that the south is home to 58% of the black U.S. population and the low acceptance of PrEP by this population [32, 37]. Among some populations, while counter-intuitive, an individual-level perceived HIV risk is not associated with a willingness to use PrEP [36, 38, 39]. On the other hand, individuals living in an area with a high area-level HIV risk are more likely to use PrEP [36]. This ‘neighborhood’ effect may generate a deeper understanding and awareness of HIV prevention, or it could be that the use of PrEP is less stigmatized in these communities. At an individual level, the perceptions of risk and PrEP uptake may depend on the timing of the risk behaviors [40]. Certainly, a factor for some high-risk individuals is the rural and low-resourced nature of much of the south. Rural areas are documented as having fewer quality primary care providers [41], in general, and perceived as less confidential and HIV-friendly specifically [42–44].

Generally, high endorsement of PrEP stigma is associated with less HIV knowledge, less willingness to use PrEP, and less perceived PrEP effectiveness [27]. Specifically, stigma beliefs that stereotype others (e.g., PrEP is for promiscuous people) are linked to less PrEP use in some populations [39, 45]. Other factors associated with acceptance of PrEP may also co-occur with an individual’s risk level. For example, among Black individuals living in the U.S., an arrest history or history of depression, an HIV positive partner, and belief in HIV conspiracies were more willing to use PrEP than Black individuals without these individual risk factors [38, 40]. Finally, a history of trauma is common in HIV populations and may be related to an individual’s adherence to treatment and risk [46, 47].

This project addressed these HIV prevention needs by examining the implementation of an HIV educational intervention in a community correctional setting in Arkansas. The study provided a preventive education intervention and offered HIV testing intended to increase individuals’ awareness of HIV intervention tools with an increased focus on the use of PrEP, therefore reducing individuals’ chances of contracting HIV. It is one of the first efforts to include PrEP as a prevention option in community legal settings. Our first goal was to examine the acceptance of preventive education in the community-based settings of adult drug courts (ADC) and probation and parole (P-P) programs by program participants. We examined the acceptance of on-site rapid HIV-testing, intent to use PrEP, and the perceived barriers to HIV testing and PrEP uptake. We also reviewed evidence that the educational intervention was useful in increasing knowledge and intentions to practice preventive health care and decreasing HIV stigma beliefs. Finally, in multivariate analyses, we examined the factors related to the intent to use PrEP and the perceived barriers to HIV testing and PrEP use.

## Methods

Eligible participants were recruited from a Community Correction Field Service (CCFS) agency in Arkansas. CCFS serves clients enrolled in Adult Drug Courts (ADC) and on probation or parole (P-P). CCFS provides various treatment services, such as alcohol and drug counselors, to treat substance abuse. Counselors provide intense supervision, monitoring, and treatment services individualized to a client’s needs and the judge’s order. All CCFS clients are subject to economic and other sanctions, frequent drug testing, and court appearances. Successful completion of the program results in dismissal of the charges, reduced or set-aside sentences, lesser penalties, or a combination of these. All ADC clients come to the facilities weekly for one or two hour-long group classes with the counselors. P-P clients have a similar schedule, but over time, required attendance may be less frequent. No cash incentives were provided; however, in support of the project, the agency allowed the time for the project-related activities to count toward required class time (e.g., allowed a day off from other activities with the counselor's approval). The human subject board approved the protocol and procedures at the University of Arkansas for Medical Sciences.

CCFS collaborated with project staff to recruit participants. Inclusion criteria were: (1) adults over 18 years of age, (2) fluent in English, and (3) enrolled in a CCFS program. CCFS agency officials provided a weekly group class schedule along with class rosters to the project staff. Counselors and the project staff jointly identified a suitable training time for each class.

### Educational Intervention

A collaboration of local researchers, a community non-profit agency addressing health literacy in HIV/AIDS, state health department professionals, and people living with HIV (PLWH) developed *Embracing Healthy Love* (EHL). The team designed EHL as a one-session, comprehensive HIV prevention educational strategy. The educational goals of EHL is to: increase HIV awareness, decrease HIV stigma, improve health practices in sexual and intravenous drug use behaviors [48]. For this project, EHL was modified to include PrEP as a prevention tool. Efforts to promote condom use include providing safe sex kits with various types of condoms and lubes, demonstrations of correct lubricant use, and education on recommendations of condoms types. The research-based content of EHL includes CDC-identified HIV/AIDS risk factors (e.g., HIV 101) and the co-occurring risks of other STIs [49]. EHL covers behavioral prevention options (e.g., condom use), biomedical treatment options (e.g., PrEP), and information on other STIs. Activities were included to engage the participants and avoid a ‘lecture’ format. These included trainer-led visualization of risk situations, short videos, demonstrations with models of use of condoms, and open-ended questions to stimulate discussion. The training lasted about an hour.

### HIV Rapid Testing

All project staff participated in a three-day HIV Prevention Voluntary Counseling and Testing Course provided by the Arkansas Department of Health. Staff training included the protocol to accurately screen for HIV using various rapid HIV tests and test results counseling. The project manager conducted HIV screening competency assessments for staff performing the rapid testing monthly. Trained project staff performed all HIV screens using the OraQuick Advance® Rapid HIV-1/2 Antibody Test (OraSure Technologies, Bethlehem, Pennsylvania). Project staff counseled participants in private.

### Protocol for Study Evaluation and Services

All ADC participants were required to attend the project educational sessions. Some probation and parole officers who worked closely with the ADC required participants to attend. The class size ranged from two to fifteen participants, with an average of 7.82 participants per class. Project staff scheduled the educational sessions multiple times to allow all participants to attend one education session. After each educational session, project staff offered participants optional HIV screens using the Rapid Test and access to Community Health Worker (CHW) support.

The CHW was assigned to facilitate health care access, including applying for healthcare coverage, obtaining and keeping medical appointments, filling prescriptions, and access/adherence to PrEP. A project-engaged, board-certified family physician was available to advocate using PrEP and provide education and consultation to community primary care providers about PrEP, including prescribing and monitoring recommendations.

Participants were informed that the study evaluation surveys and CHW support were optional. Project staff provided participants with a random-generated I.D. number. The number was used to link the participant to all study data. Before EHL, participants reported basic demographic, HIV knowledge and completed a risk assessment survey.

Post-intervention, project staff counseled one-on-one with participants who accepted free HIV testing and explained the testing process and confidentiality of information and results. Participants could either wait 20 min to receive test results in person or receive a phone call from the staff member. Participants who wished to receive a phone call from the staff member would establish a four-digit password to confirm their identification during the phone conversation. If the HIV test was non-reactive, participants received informational sheets and consultation for PrEP as a preventative measure. If the HIV screen was reactive, project staff counseled participants and provided a referral for a confirmatory test and linkage to treatment services and support. Per agreement with the ADC judge, all ADC participants who accepted HIV testing were incentivized by being excused from one future ADC group class.

### Measures

#### Assessment of Correlates of Outcomes

##### Barriers to Care

Participants rated three areas to assess barriers [50]*,* as shown in Table 1. Access to insurance was considered a risk if the participant reported being uninsured. Government insurance included those with V.A. benefits, Medicare, or Medicaid. Participants who indicated they would have issues being able to pay medical co-pays or take off work to get medical care were also considered to have barriers (1 item each with yes/no response options). Barriers were summarized as the number of barriers reported (score from 0 to 3).

**Table 1:** Demographic characteristics of the sample (N = 218)

Variables	Adult drug court (ADC) N = 123	Probation & Parole (P&P) N = 95	Total N = 218
Age years—mean (SD)	39.98 (n = 117, SD = 10.97)	37.74 (n = 88, SD = 10.05)	39.02 (SD = 10.62)
Marital status	% (n)^a^	% (n)^b^	
Single/never married	27.6% (34)	41.1% (39)	33.5%
Married/committed	48.0% (58)	37.9% (94)	43.6%
Widowed, separated, divorced	24.4% (31)	21.1% (85)	22.9%
Gender**			
Male	56.1% (69)	75.8% (72)	64.7%
Female	42.3% (52)	23.2% (22)	33.9%
Transgender	1.6% (2)	1.1% (1)	1.4%
Race**			
White	55.3% (68)	29.5% (28)	44.0%
Black/African American	36.6% (45)	66.3% (63)	49.5%
Other	8.1% (10)	4.2% (4)	6.4%
Education level**			
Did not complete high school	8.1% (10)	21.3% (20)	13.8%
High school graduate/GED	40.7% (50)	50.0% (47)	44.7%
Some college	36.6% (45)	25.5% (24)	31.8%
College degree or higher	14.6% (63)	3.2% (27)	9.7%
Barriers to medical care			
Type of insurance*			
Private insurance	43.1% (53)	27.4% (26)	36.2%
Government insurance	48.0% (59)	52.6% (50)	50.0%
None	8.9% (11)	20.0% (19)	13.8%
Copay a barrier**	17.5% (21)	35.8% (34)	25.6%
Time from work a barrier	12.6% (15)	12.2% (11)	12.4%
Number of barriers—mean (N, SD)**	0.38 (123, 0.62)	0.67 (95, 0.85)	0.51 (0.74)
Personal history			
Trauma history	59.8% (70)	58.1% (54)	59.0%
Depressive symptoms	30.0% (36)	36.2% (34)	32.7%

*p < .05, **p < .01

^a^n is the numerator for the percent, the denominator ranged from 117 to 123

^b^n is the numerator for the percent, the denominator ranged from 88 to 94

##### Personal History

Participants rated their level of depressive symptoms (see Table 1) with the PHQ-2 [51]. The two questions (e.g., *“little interest or pleasure in doing things”*) targeted the last two weeks and were assessed on a 4 point scale (0 = not at all to 3 = nearly every day). The PHQ-2 score is the sum of the two items and ranges from 0 to 3, with a score of 3 or more indicating major depressive disorder is likely.

A history of trauma was assessed with one item (e.g., “… *experienced violence or trauma in any setting*…”) with a range of trauma examples provided. Trauma history was noted if the participant agreed or strongly agreed.

##### HIV Risk

The assessments of behaviors related to HIV risk are shown in Table 2. The assessment included one global question of perceived risk (i.e., “*Do you feel that you are at risk for HIV*?”) and five questions of life-time events. These included: ever been in jail, used a needle to inject, shared equipment, exchanged sex for something of value, or had a sexually transmitted disease [52, 53]. Response options were yes or no.

**Table 2:** Risk indicators, stigma, and knowledge of the sample (N = 218)

	Adult drug court (ADC)	Probation & parole (P-P)	Total
HIV risk indicators	% (n)^a^	% (n)^b^	
Feel that you are at risk for HIV?	6.6% (8)	12.2% (11)	9.0%
Alcohol and drug use in last year			
Alcohol to intoxication	35.1% (33)	49.3% (34)	41.1
Alcohol and drugs same day**	23.9% (21)	46.2% (30)	33.3
Illegal drug use—non-IV	45.5% (56)	43.2% (41)	44.5
Drug—injection related			
Ever used a needle to inject drugs?**	34.4% (42)	13.3% (12)	25.5%
Shared equipment**^, PrEP^	22.0% (27)	6.7% (6)	15.5%
Sex related risk^c^	% (n)^d^	% (n)^e^	
Exchanged sex for something of value	20.5% (25)	16.7% (15)	18.9%
Ever had STD^PrEP^	30.6% (37)	34.4% (31)	32.2%
Multiple partners*	31.3% (35)	46.3% (38)	37.6%
Partners HIV + **^, PrEP^	3.7% (4)	16.5% (13)	9.1%
SEX with IV user^PrEP^	14.0% (14)	15.2% (12)	14.5%
Use of condoms^PrEP^	75.8% (75)	69.7% (53)	73.1%
Anal sex^F^	48.4% (45)	57.4% (39)	52.2%
Total with any HIV risk	92.7% (114)	88.4% (84)	90.8%
Total risk indicated for PrEP	74.0% (91)	75.6% (68)	74.6%
Total risk for HIV—mean (N, SD)	3.67 (2.41)	4.00 (2.88)	3.81 (2.63)
Total risk indicated for PrEP- mean (N, SD)	1.28 (123, 1.11)	1.28 (90, 1.10)	1.28 (1.11)

*p < 0.05, **p < 0.01

^PrEP^Strongest recommendations for PrEP by the CDC

^a^n is the numerator for the percent, the denominator ranged from 122 to 123

^b^n is the numerator for the percent, the denominator ranged from 90 to 94

^c^24 respondents left this blank and 33 indicated no sexual relations in the last year

^d^n is the numerator for the percent, the denominator ranged from 112 to 123

^e^n is the numerator for the percent, the denominator ranged from 81 to 94

^f^The denominator ranged from 93 to 68

Assessment items related to sexual or drug-related risks were based on the last year [54]. Sexual risk included the number of sexual partners, having sex with PLWH or IV drug users, and sex (anal or vaginal) without condoms. More than one sexual partner was considered a risk. A response of yes or unknown sexual relations with PLWH or IV drug users was considered a risk. Sexual relations without a condom ‘always’ was considered a risk. Alcohol and drug use in last year was assessed with a detailed survey of 19 illegal drugs (e.g., heroin, methamphetamine) or misuse of legal drugs (e.g., Opioids like Oxycodone, Morphine) and five methods of use (e.g., oral, IV). An additional four items focused on the misuse of alcohol (e.g., *alcohol to intoxication*). These were summarized as shown in Table 1. Alcohol to Intoxication was a yes response to two items. Alcohol and Drugs Same Day was a yes response to one item. Illegal Drug Use – Non-IV was a yes response to any of the 19 drugs in any mode except IV. Illegal IV Drug Use was created with a yes response to any of the 19 drugs only if IV was indicated.

##### HIV Risk/PrEP

The CDC recommends PrEP for those with specific sexual and illegal drug use behaviors [55]. These items are marked in Table 2 with a superscript. Five of these were assessed and included: sharing IV equipment, STI history, sexual partners with HIV or/and IV user, and inconsistent use of condoms. This risk was operationalized as the sum of these variables as previously described resulting in a score from 0 to 5 with higher scores indicating more risk.

### Assessment of Usefulness of Educational Intervention

#### Acceptance and Usefulness of Intervention

Participants rated the ratings of EHL trainer and sessions (see Table 3) with a 7-item questionnaire addressing: the usefulness of the training (1 item, ‘*the education useful’*), the quality of the trainer’s knowledge (4 items, e.g.,*‘ the presenter knowledgeable about prevention of HIV’*) and skill (2 items, e.g., *‘feel comfortable asking questions’*). Ratings were on a five-point Likert scale (1 = poor, 5 = excellent). The items had high reliability (alpha = 0.95) and were averaged to obtain a summary score with higher scores indicating more support for EHL.

**Table 3:** Evidence of *Embracing Healthy Love* (EHL) and project activities acceptance and usefulness- overall and by group (N = 218)

	Adult drug court (ADC) % (n)^a^	Probation & parole (P&P) % (n)^b^	Total
Usefulness of EHL educational intervention			
Ratings of EHL sessions—mean (N, SD)^c^	4.46 (119, 0.74)	4.58 (90, 0.71)	4.51 (0.73)
Acceptance of HIV testing	33.0% (123)	40.0% (93)	36.0%
Interest in PrEP	27.9% (29)	42.5% (34)*	34.2%
HIV stigma beliefs^d^			
Pre beliefs—mean (SD)	2.13 (113, .52)	2.24 (89, .55)	2.18 (.53)
Post stigma beliefs—mean (SD)	2.09 (113, .55)	2.24 (89, .52)	2.15 (.54)
HIV knowledge^e^			
Pre education % correct**	77.1% (n = 123)	72.1% (n = 94)	74.6%
Post education % correct	81.4% (n = 123)	80.7% (n = 94)	81.1%

*p < 0.05, **p < 0.01

^a^n is the numerator for the percent, the denominator ranged from 113 to 123

^b^n is the numerator for the percent, the denominator ranged from 89 to 94

^c^High scores positive view of content, climate, and trainer, out of 5

^d^High scores indicate more stigma beliefs, out of 4

^e^High scores indicate percent knowledge correct

##### HIV Knowledge

Six types of behaviors linked with HIV risk were assessed [56]*.* Yes or no responses were rated on 23 items. Items were grouped in content related to HIV testing (1 item), HIV transmission behavior (8 items), transmission fluids (6 items), prevention (6 items), treatment (1 item), and appearance (1 item). Sample items include *“With treatment, can you live a long and healthy life with HIV?”* (treatment), *“HIV is spread through … blood”* (transmission fluids), *“HIV is spread through …giving birth”* (transmission behavior). The correct scores were summed with high scores indicating correct responses (See Table 3).

#### HIV Stigma Perception

Based on the HIV Stigma Framework proposed by Earnshaw et al. [57], three items were selected to represent three aspects of stigma: prejudice, stereotype, and discrimination. Each was rated on a 4-point Likert scale (1 = strongly disagree, 4 = strongly agree). Stereotype was assessed with the item ‘*Most people who are HIV positive do not care if they infect other people*.’ Prejudice was assessed with the item ‘*I would feel ashamed if someone in my family was HIV positive*’. Discrimination was assessed with the item “*Women who are HIV positive should be allowed to bear children if they wish* (reverse scored).” The items had low correlation with each other (r less than 0.11), suggesting assessment of different constructs. The responses were summed so that high scores indicate more stigma beliefs, with scores shown in Table 3.

### Outcome Variables

#### Acceptance of HIV Testing and Intent to use PrEP

Acceptance of HIV testing was assessed in the post-survey with a single item (e.g., “*would you like to obtain HIV Rapid Test screen for HIV status?*”). Response options were yes/no. PrEP interest was recorded by a single question “*How likely is it you will ask your doctor about Truvada for oral pre-exposure prophylaxis (PrEP) for your use*?” on a four-point Likert scale (1 = not very likely, 4 = very likely). The score was summarized such that ‘not very’ and ‘not likely’ were combined as not interested and ‘likely’ and ‘very likely’ as interested (see Table 4).

**Table 4:** Bivariate examination of predictors of intent to use PrEP

	Intent to use Prep (N = 63) % (n)^a^	No intent to use Prep (N = 121) % (n)^b^	Total
HIV risk behavior ^PrEP^			
Shared equipment*	16.9% (20)	6.3% (4)	13.3%
Ever had STD	29.7% (35)	38.7% (24	32.8%
Partners HIV	8.7% (4)	7.5% (9)	8.3%
Sex with IV user	15.8% (5)	9.8% (16)	13.8%
Use of condoms	31.5% (35)	68.5% (76)	74.5%
Number of risks—mean (SD)	1.14 (0.98)	1.32 (1.01)	1.28 (1.11)
Demographic			
Age (N, SD) +	40.9 (59, 10.1)	37.7 (115, 10.8)	38.8 (10.6)
Marital status – with partner/married	44.6% (54)	41.3% (26)	43.5%
Race*			
White	52.1% (20)	31.7% (63)	45.1%
Black/African American	41.3% (40)	63.5% (50)	48.9%
Other	6.6% (3)	4.8% (8)	6.0%
Education level			
Did not complete high school	11.7% (10)	15.9% (14)	13.1%
High school graduate/GED	43.3% (26)	41.3% (52)	42.6%
Some college	32.5% (24)	38.1% (39)	34.4%
College degree or higher	12.5% (3)	4.8% (15)	9.8%
Barriers to medical care			
Type of insurance			
Private insurance	38.8% (23)	36.5% (47)	38.0%
Government insurance	47.1% (35)	55.6% (57)	50.0%
None	14.0% (5)	7.9% (17)	12.0%
Copay a barrier	76.3% (47)	74.6% (90)	75.7%
Time from work a barrier	14.2% (7)	12.1% (17)	13.5%
Number of barriers—mean (N, SD)	0.44 (63, 0.64)	0.51 (121, 0.79)	0.49 (0.74)
Personal history			
Trauma history	59.8% (38)	57.1% (70)	58.9%
Depressive symptoms—mean (N, SD)**	2.11 (63, 0.72)	1.77 (119, 0.78)	1.89 (0.77)
HIV stigma beliefs			
Stereotypes*	31.3% (32)	48.4% (36)	27.3%
Prejudice*	4.3% (8)	12.9% (5)	7.3%
Discrimination	51.8% (33)	53.2% (59)	52.3%
Stigma beliefs—mean (N, SD)*	6.9 (61, 1.5)	6.3 (110, 1.5)	6.4 (1.6)
HIV knowledge % correct	84.5% (63)	84.3% (121)	84.3%

+ p < 0.1, *p < 0.05, **p < 0.01

^PrEP^Strongest recommendations for PrEP by the CDC

^a^n is the numerator for the percent, the denominator ranged from 59 to 63

^b^n is the numerator for the percent, the denominator ranged from 114 to 121

### Statistical Analyses

Analyses were conducted using SPSS statistical software (Version 26). Group differences were examined using chi-square statistics or independent t-tests. Paired t-test was used to examine continuous scores across time. Logistic regression was used to examine the factors associated with intent to use PrEP.

## Results

The sample included 218 adults recruited from a CCFS agency located in an urban area of a southern U.S. state. Recruited clients included those in ADC (n = 123) and on P-P (n = 95) as described in Table 1. Participants were recruited between September 2018 and January 2019. Table 1 shows the demographic characteristics of participants by legal status. The mean age was 39.02 years old (n = 205, SD 10.62). Current relationship status was reported as following: 33.5% as Single/Never Married, 24.3% as in a Committed Relationship but not Married, 18.8% as Married, 16.5% as Divorced, 1.4% as Windowed, 4.1% as Separated, 1.4% as Other.

Overall, 64.7% of the participants were cis-male, 33.9% were cis-female, and three were transgender. However, P-P participants were more likely to be males (75.8% vs. 56.1% ADC participants). Overall, the majority of the participants identified as non-Hispanic White (44.0%) and non-Hispanic African American (49.5%). Fourteen (14) either selected a race of ‘other’ or selected Hispanic (n = 3), American Indian/Alaskan Native (3), or Asian (1) P-P participants were more likely to be African American (66.3%) compared to ADC participants (55.3%).

The majority of participants were high school graduates, GED or equivalent (44.7%), or some college (31.8%); however, 13.8% did not complete high school, and 9.7% had completed a college degree or higher. P-P participants were more likely to be high school dropouts (21.3% compared to 8.1% ADC participants), and ADC participants were more likely to have a college degree than P-P participants were.

Many participants reported a history of trauma (59%), and about a third (32.7%) reported depressive symptoms. P-P participants reported more barriers to care, including 20% with no insurance—public or private—compared to only 8.9% of ADC participants. P-P participants were also less likely than ADC participants to have the ability to provide medical co-pays.

As expected, based on the recruitment population, nearly all reported being incarcerated at some point (96.7%). As shown in Table 2, many participants reported behaviors related to HIV risk. In some areas, the participants in both ADC and P&P reported similar risk behavior. For example, most in both groups reported inconsistent use of condoms (73.1%), sex with an IV drug user (14.5%), and exchanging sex for something of value (18.9%). However, in other areas, the risks were different. More ADC participants were likely to report drug use by IV route and shared equipment than P-P participants. However, more P-P participants reported having multiple partners and a partner who was PLWH than ADC participants did.

### Acceptance and Usefulness of Intervention

Tracking records from EHL showed that 299 participants were directed to the sessions, and 218 attended (73%). The average class size was eight people. All participants rated the training and trainers high (M = 4.51, SD 0.73) with no significant difference between ADC and P&P, as shown in Table 3. Ratings on all seven items were above 4 on a 5-point scale. Open-ended questions asking for participants’ feedback on the training were overwhelmingly positive such as “*Please keep educating people and testing*,” “*Thanks for the knowledge*,” and “*I appreciate this class—very informative*”.

Over a third (36.0%) indicated an interest in HIV testing at the end of the session across groups. Among the participants who declined HIV testing, the most common were “*I know my HIV status*” (36.1%), “*I don’t believe that I’m at risk*” (25.9%), and “*I don’t have time but would like it another day*” (10.2%). This last group was offered information to obtain the test later.

Three (n = 3) P-P participants indicated they were currently using PrEP. Overall, 34.2% of participants expressed interest in asking their doctors about PrEP. Significantly more P-P participants expressed interest in PrEP than ADC participants (42.5% compared to 27.9% of ADC, (*X*^2^ (1, *n* = 184) = 4.29, *p* = 0.04)). Among the participants who indicated no interest in PrEP, the most common reasons were “*I don’t think I’m at risk*” (39.8%), “*I don’t think I need it in addition to condoms*” (4.6%), and “*I don’t like to take medicine*” (4.2%).

### HIV Prevention Effect of EHL

As shown in Table 3, HIV stigma scores were not reduced by posttest based on paired t-tests. HIV knowledge increased from pre-education to post with a 6.5% increase in correct answers for all participants (t (216) = − 0.6.26, p = 0.00). Follow-up analyses by group indicated that increases were statistically significant in both groups. In a Univariate Analyses of Variance, group membership was not significant in predicting post score change while controlling for pre-test score (group: F(1,214) = 1.96 p = 0.16).

### Factors Related to Intent to Use PrEP

Table 4 shows the bivariate relationships between intent to use PrEP and an array of participant characteristics: HIV risk, demographic characteristics, barriers to medical care, personal history characteristics, stigma beliefs, and HIV knowledge. Among the five behaviors, CDC identifies as indicators that PrEP should be recommended, only one is linked with interest in PrEP. Of individuals reporting that they shared intravenous equipment, nearly three times as many were interested in PrEP than not (*X*^2^ (1, *N* = 181) = 4.01, *p* > 0.04). With this one exception, increased self-report risk behavior was not related to increased interest in PrEP.

Of the demographic characteristics, white race was linked to interest in PrEP (42.1% interested vs 31.7% not interested, (*X*^2^ (2, *N* = 184) = 8.19, *p* = 0.02) and Black race was linked with less interest in PrEP (41.3% vs 63.5%). Individuals that reported more depressive symptoms were more likely to be interested in PrEP (t (181) = 2.86, p = 0.005). Finally, specific types of HIV stigma belief was associated with disinterested in PrEP (t (171) = 2.51, p = 0.03). Specifically, stereotyping (*X*^2^ (1, *N* = 177) = 5.03, *p* = 0.02) and prejudice beliefs (*X*^2^ (1, *N* = 179) = 4.49, *p* = 0.03) were higher among those not interested in PrEP.

Based on the bivariate analyses shown in Table 4, we examined a multivariate model with all factors shown in Table 5 with a bivariate relationship to intent to use PrEP of at least p = 0.1. Using logistic regression, we predicted intent to use PrEP from six factors: group (ADC vs. P-P), race, age, level of depressive symptoms, sharing IV materials, and two stigma beliefs (stereotyping, prejudice). The model fit well (X^2^ (8, N = 164) = 31.10, p = 0.00). In this multivariate model, three factors were statistically significant predictors: prejudice stigma beliefs (t(1) = 4.77, p = 0.03), depressive symptoms (t(1) = 7.27, p = 0.00), and race (t(1) = 6.82, p = 0.03).

**Table 5:** Logistic regression with bivariate predictors of intent to use PrEP

	B	SE	aOR
Group (ADC vs P-P)	− 0.56	0.39	0.57
Stereotyping stigma beliefs	− 0.08	0.24	0.93
Prejudice stigma beliefs*	0.63	0.29	1.87
Age	0.03	0.02	1.03
Depressed**	0.72	0.27	2.04
Sharing IV materials	0.41	0.66	1.51
Race			
Other	0.86	0.81	2.37
Black*	1.13	0.44	3.11

Omnibus test of model fit (X^2^ (8, N = 164) = 31.10, p = 0.00), White is the referent group for Race comparisons

*SE* standard error, *aOR* adjusted odds ratio

*p < 0.05, **p < 0.01

Interpreting the adjusted odds ratios (aOR), each increase in agreement (e.g., Disagree to Agree) with prejudice stigma beliefs is associated with increased odds of nearly two that the individual would reject PrEP (*aOR* = 1.87, *CI* [1.07, 3.29], *p* = 0.03). Alternatively, a one-point increase in depressive symptoms is associated with slightly more than double the odds (*aOR* = 2.04, *CI* [1.22, 3.44], *p* = 0.007) of accepting the use of PrEP. Finally, Black participants had odds that were more than three times (*aOR* = 3.11, *CI* [1.31, 7.36], *p* = 0.01) those of white participants to endorse rejecting PrEP as an option.

## Discussion

Adult Drug Court (ADC) Best Practice Standards encourage interventions to reduce HIV among ADC enrollees, yet very few ADCs address HIV prevention [9, 10, 13]. HIV prevention education and support for HIV testing are far from universal in these high-risk populations [58]. This study provided evidence to support the acceptance and usefulness of an HIV education intervention, *Embracing Healthy Love* (EHL), for ADC and P-P program participants. Program participants rated the training high as a useful and accepting climate. EHL presenters were rated as knowledgeable and engaging. Participants scored higher on HIV knowledge after attending the EHL training. Participants' knowledge scores moving from mid-to-low C grades to low B grades. The baseline level of stigma was high, with nearly 50% of participants indicating agreement with at least one area of stigma beliefs. However, sigma beliefs did not improved post training. That is, it appears that increasing knowledge did not reduce the sigma beliefs of participants.

Program staff worked in partnership with the project staff to overcome a range of barriers (e.g., a physical move of the program into a new building), suggesting they valued the project. Study staff tracked reactions and events related to the acceptance of the project by CCFS agency staff members. Several unusual events occurred, but the CCFS staff always worked with project staff to find solutions while disruptive. For example, the ADC intake was temporarily closed due to a change in the judge for the court. The CCFS agency leadership changed and required additional logistic adjustments. Lastly, the CCFS program moved into a smaller space causing difficulty finding space for informational sessions and space for the CHW. With each new disruption, the CCFS agency staff worked with the project staff resulting in the project ending with the expected training targets.

Nearly all ADC and P-P participants self-reported risk behaviors linked to HIV acquisition; however, the types of behaviors differed. ADC participants more often reported drug-related behaviors (e.g., sharing equipment and using IV drugs) compared to P-P participants. P-P participants reported more sex related risks that ADC participants. Among the behaviors listed in the CDC recommendations warranting the use of PrEP, about 75% of participants reported at least one behavior. After educational intervention, only about a third (34.2%) indicated intent to use PrEP and only 36% accepted free, on-site HIV testing. The disconnect between self-reported risks and recognition of risk has been documented in other studies of HIV-negative individuals but is not universal [36, 38–40].

This study examined a range of factors thought to be predictive of intent to use PrEP, such as access to medical insurance, HIV knowledge, age, and race. In a multivariate examination of these factors, only three factors were predictive in this population's justice system. First, increased depressive symptoms were linked with more interest in the use of PrEP. This may be explained by increased sexual risk-taking with depression [59]. However, in this study, increased risk behavior was not, in general, predictive of an intent to use PrEP in bivariate or multivariate analyses. Depression is thought to inhibit many individuals from self-care; however, not in the case of interest in PrEP. Others have found that depression did not reduce adherence to PrEP use [60]. That is, depression did not prevent the proactive behaviors of seeking and adhering to HIV prevention.

Second, we found that Black participants were much less likely to indicate an intent to use PrEP than white participants. A general mistrust of established medical care by minorities may drive this in the government regarding medical issues. The distrust by Black participants has been documented in other studies on a range of medical conditions (e.g., colorectal cancer screening) and HIV testing and treatment specifically [61–63]. Unfortunately, a cultural history that includes oppression and discrimination provides an ample mix of truth and fiction to cause general confusion and distrust.

Finally, the stigma around HIV has long been a barrier to treatment for PLWH; however, it is also harmful to people of unknown status [64]. Earnshaw and Chaudoir’s [57] framework for HIV stigma and reduced testing was based on the concept that people with unknown status made efforts to distance themselves from the ‘others’. Adopting stigmatizing beliefs such as stereotyping or prejudice towards people living with HIV or likely to have HIV provides this separation. In this study, controlling for other factors, prejudiced belief were a barrier to acceptance to PrEP. Stigma-related concerns about PrEP have been noted as barriers to PrEP use. Unfortunately, few interventions have been tested or shown success in changing prejudiced attitudes [65, 66]. However, some hopeful evidence suggests that increased knowledge is less helpful than more personal and one-on-one [67].

Perhaps, it should not be surprising that this short educational intervention did not result in a change in stigma attitudes. When increased knowledge is linked to changes, it is at best modest in size [68]. Further, in the situation examined in this study, the culture of the south may be making change even more difficult. The stereotype that the Southern US states are extremely religious is deserved. For example, the south is the home to the largest groups of Black protestants and white evangelical protestants [69] in the country. While the teachings of religious groups vary, the stance of large religious groups in the south can hardly be viewed as accepting of homosexual behaviors [70]. Still, the influence of religion on stigma beliefs depends on ‘specific beliefs, attitudes, and practices found within the community [71] and therefore varies even in the south. Regarding substance use disorders, stigma toward those with this disorder is widespread [72]. Finally, in this study, all participants were offenders. They were likely factoring in their perception of being stigmatized regardless of their additional risk of being included in other risk categories (i.e., behaviors linked to HIV).

Understanding the balancing by participants of their reported behavior, acknowledging their risk of HIV acquisition, their access to highly effective prevention options, and the risk of being viewed as the target of ‘others’ by their community is critical in the development of interventions in the south. This is a dynamic balance as the broader culture in the U.S. becomes more tolerant and accepting of homosexuality and influences Christian leaders [73]. Further, interventions may need to include a more comprehensive community component by engaging with religious leaders or addressing the stigma with concurrent and targeted media campaigns. A commonly accepted approach to combating stigma beliefs is to provide ‘intergroup’ contact. For example, testing the differential effect of EHL classes led by trainers that are HIV positive compared to trainers that are not.

This study has several limitations. First, like many studies, it relies primarily on self-report. Second, we measured the intent to use PrEP. While this is an first step, we could not assess the follow-through of this intent. Third, the sample may not be representative of ADC and P-P individuals at a national level. On the other hand, attendance and participation in the evaluation were high. Fourth, there were multiple disruptions to the delivery of the program because of changes at the program level. However, the willingness of program staff to find solutions speaks well of the acceptance of the intervention efforts. It is possible the disruption dampened the positive findings. Finally, to keep our evaluation survey short, we included only one item for each type of stigma belief. Unfortunately, this did not allow us to assess reliability.

The strengths of this study included targeting a high-risk population and support for a useful and acceptable educational intervention. The finding that access to medical care was not the primary barrier to the refusal to consider PrEP is useful. Understanding the specific focus (e.g., Sterotype and Prejudice) of the stigmatizing beliefs will help tailor educational interventions to address this barrier in future intervention development.

High rates of HIV/STI substance misusers contribute to high rates of HIV infection among individuals in the criminal justice system, including community settings. Substance misusers often engage in risky behaviors, such as sharing needles. Further, drug use can lead to unprotected sex, potentially with multiple partners or sex with other substance misusers, that put them at risk for HIV/STDs [11]. Adult drug courts and systems serving individuals on probation and parole may provide a window of opportunity to deliver HIV/STI prevention programming [10]. Given the high risk documented for ADC and P-P individuals, HIV prevention is a critical component for increased protective behaviors.
